# The Influence of Social Support and Social Integration Factors on Return to Work Outcomes for Individuals with Work-Related Injuries: A Systematic Review

**DOI:** 10.1007/s10926-018-09826-x

**Published:** 2019-01-22

**Authors:** Codi White, Rebecca A. Green, Samantha Ferguson, Sarah L. Anderson, Caroline Howe, Jing Sun, Nicholas Buys

**Affiliations:** 1 Griffith University, Gold Coast, Australia; 2 Insurance and Care NSW, Sydney, Australia

**Keywords:** Social support, Return to work, Occupational injuries

## Abstract

*Purpose* In occupational rehabilitation, the biopsychosocial model endorses the role of social factors in worker recovery. We conducted a systematic review to explore three questions examining the role of social support for the return-to-work (RTW) of individuals with work-related injury: (1) What are the worker-identified social barriers and facilitators in RTW; (2) What is the relationship between social factors and RTW; and (3) What is the effectiveness of social interventions for RTW. *Methods* Systematic searches of six databases were conducted for each research question. These identified 11 studies meeting inclusion criteria for Research Question 1, and 12 studies for Research Question 2. No studies were identified that met inclusion criteria for Research Question 3. A narrative synthesis approach was used to analyse the included studies. *Results* Research Question 1 identified five themes in social barriers and facilitators to RTW, including *contact*/*communication, person-centred approaches, mutual trust, reaction to injury*, and *social relationships*. Research Question 2 identified moderate support for *reaction to injury* and *social integration*/*functioning* as predictors of RTW and weak evidence for *co-worker support*. Four studies reported significant associations between social factors and RTW, six reported mixed findings with at least one significant social predictor, and two found no significant relationships. However, conclusions were limited by the inconsistency in measurement of social factors. *Conclusions* Our findings indicate that social support and integration may influence RTW following work-related injury, and highlights the need for further systematic examination of social factors in the field of occupational rehabilitation.

Although the biopsychosocial model is considered best practice in the field of occupational rehabilitation, the “social” component of this model is often neglected [1]. This is problematic as previous research has shown that social factors continue to play an important role in injured workers return-to-work (RTW) process, including workers being at increased risk of secondary psychosocial impairment (e.g., depression, disruption to roles or relationships) when they receive inappropriate care or insufficient support [2–4]. In fact, social and relationship factors have been shown to be important for the prevention of injury and illness generally, with factors such as social relationships, family ties, and greater social contact, showing protective effects on mortality and morbidity [5, 6]. Further, when examining preventative strategies for managing non-compensable sickness absence, social factors like supervisory support and leadership quality were effective in reducing absences and increasing productivity [7–9], while interventions targeting social support and supervisory quality have been found to increase the risk of work absence and improve productivity [10].

According to social capital theory [11], the social resources of an individual are critical to their ability to cope with external stressors, such as recovering from an injury or illness. These social resources comprise individuals access to social support [12, 13] (i.e. perceived or actual help offered from non-professional others), and their social integration [14], or the extent to which they engage with different relationships and perceive themselves to belong to different communities. However, despite mounting evidence of the importance of social factors for recovery and wellbeing, there is still little consensus regarding their role for workers who have become injured or ill as a result of their work (i.e. work-related injuries). Work-related injury or illness is broadly defined as any psychological or physical harm sustained in the course of one’s work duties [15, 16]. Research has found that injured workers with compensable work-related injuries or illnesses fare more poorly in their recovery and RTW outcomes [17] and it has been suggested that this is due to additional social obstacles to RTW (e.g., social pressure or isolation from connections at work), that occur primarily when an injury or illness is work-related [2, 3].

Currently, research that has examined the influence of social factors in compensable injuries or illnesses has focused primarily on interactions between the worker and their employer, insurer, or healthcare providers [4, 18–23]. However, there has yet to be a systematic examination of the influence of social support and social integration for injured workers’ workplace (e.g., supervisor, co-workers) and personal social connections (e.g., family, friends) on the RTW process [12, 24, 25]. Given the strong links found between social factors and health and wellbeing in past research, the current paper seeks to address this gap by providing a systematic examination of the available literature examining the influence of social support and social integration from work (e.g., co-workers, supervisors) and non-work (e.g., family, friends, wider community) contexts on the RTW outcomes of workers with work-related injuries. Therefore, this systematic review addresses three review questions:

1. Which elements of the social context are identified by injured workers as important facilitators of, or barriers in, their RTW?
2. Is there an association between social support and social integration (within and outside the workplace) with the RTW outcomes of injured workers?
3. Are interventions focused on social support and social integration in work and non-work related contexts effective in increasing RTW outcomes for individuals with work-related injuries?

## Method

### Protocol and Registration

The current review was registered on Prospero (CRD42018086954), available at http://www.crd.york.ac.uk/PROSPERO/display_record.php?ID=CRD42018086954. A full protocol was developed and is available from the authors upon request.

### Eligibility Criteria

The PICO structure [e.g., 26, 27] was utilised to determine the inclusion of studies using the: population (P), intervention (I), control (C), and outcome (O).

#### Population

Studies were included when at least 70% of participants (or 70% of a subsample of participants) met the following eligibility criteria as described within the article: (1) participants were adults of working age (18–65 years) with a work-related injury or illness (i.e. a physical or psychological harm that occurred at or was attributed to work); and (2) participants were employed at the time of injury/illness and were seeking to return to the same workplace. Studies were excluded when the primary participant pool was drawn from military personnel, sports players, or self-employed workers. As the social consequences of a work-related injury should be comparable across different types of injury (e.g., slips, trips, falls, musculoskeletal disorders, or other work-related incidents that cause injury or illness), this review did not use injury type as an inclusion/exclusion criteria, except where the harm caused was severe enough to make return to the same pre-injury job unlikely (i.e. spinal cord injury, traumatic brain injury, burn injuries to > 30% of body, sensory loss > 50%, amputation > 30%). As social factors are often neglected within the rehabilitation management field, it was considered pertinent to capture social factors that emerged from diverse injury and illness populations and it was expected that regardless of the injury type experienced, those with work-related injuries seeking to return to their place of employment would face similar social factors.

#### Indicator/Intervention

For inclusion in Review Question 1 or 2, studies were required to examine the impact of social support and social integration on RTW for individuals with work-related injuries. This includes perceived and actual social support from supervisors, co-workers, family, friends, peers and mentors, as well as the cognitive (e.g., sense of community, belonging, and isolation) and behavioural (e.g., engagement in social activities and interpersonal relationship quality) components of social integration. For inclusion in Review Question 3, studies were required to include an evaluation of the effectiveness of a RTW intervention that aimed to increase social support and/or social integration for workers who had received a work-related injury. Social factors could also be included within a multifaceted intervention, provided that the social element involved direct intervention and was a key and standardised component of the intervention that was administered to all participants. Further, for all three reviews, studies that focused solely on relationships with a healthcare provider (e.g., doctor, rehabilitation worker, or occupational therapist) or insurance representative were excluded.

#### Control

For Review Question 1 and 2, no control condition was required. For Review Question 3, studies were required to have a care as usual control condition for inclusion.

#### Outcome

Review Question 1 addressed qualitative studies, with classification of social factors as facilitators or barriers in the RTW process the outcomes of interest. For this review question, social factors had to be identified by the injured worker. Findings of social factors identified by other stakeholders (e.g., employers, rehabilitation providers, family) were not included in the current review. Review Questions 2 and 3 examined quantitative studies, with RTW status and timing as the outcomes of interest. As the measurement of RTW varies substantially between studies, all RTW status (e.g., partial, full, or sustained RTW [SRTW]) and RTW timing (e.g., duration of absence/compensation) outcomes were included. Table 1 provides details of how RTW was assessed in each study, including the length of follow-up from time of injury where available [28–50].

**Table 1:** Characteristics of included studies

Review 1: facilitators of and barriers to return-to-work (qualitative studies)														
Study		Country		Sample size		Population; type of injury/illness		Method of data collection		Research focus				
Bunzli et al. 2017 [28]		AU		93		Compensable musculoskeletal or psychological injury; 71% male; mean age, 48 years		Individual interviews (semi-structured)		Influence of the wider social context on injured workers’ fear of (re)injury and RTW behaviour				
Buys et al. 2017 [29]		AU		17		Non-specific work-related injury or illness; 41.1% male; (age: 35–64 years)		Group interviews (semi-structured)		The relationship between disability management and organisational culture in Australian and Canadian organisations				
Cheng et al. 2011 [30]		CN		12		Non-specific work-related injury or illness; 75% male; mean age in years (SD), 34.77 (4.94)		Focus groups		The views of key RTW stakeholders on necessary activities for RTW coordination				
Kosny et al. 2012 [31]		CA		9		Non-specific work-related injury or illness; all male; (Age: 40–60+ years)		Focus groups		The role that co-workers play after a work-related injury and during the RTW process in the unionized, electrical construction sector				
Lysaght et al. 2008 [32]		CA		18		Non-specific work-related injury or illness; 22.2% male; mean age, 47.7 years		Individual interviews (open)		Workplace disability support				
MacEachen et al. 2007 [33]		CA		37		Non-specific work-related injury or illness; 62.2% male; (age: 30–69 years)		Individual interviews (semi-structured)		Injured worker peer support groups				
Mansfield et al. 2014 [34]		CA		13		Electrical workers who have experienced an electrical injury at work; all male		Individual interviews (semi-structured)		Social, institutional, and relational elements that workers perceived to influence RTW				
Mullen et al. 2015 [35]		US		16		Nurses who have experienced work-related musculoskeletal pain/disorders; all female; mean age in years (SD), 51.5 (7.4)		Individual interviews (semi-structured)		Nurses perspectives of obstacles and motivations to return to work				
Norland et al. 2013 [36]		SE		12		Work-related exhaustive disorder (burnout); 16.7% male; mean age, 39 years		Individual interviews (semi-structured)		Experiences and thoughts in the process of RTW				
Soklaridis et al. 2010 [37]		CA		6		Work-related back pain		Focus groups		Psychosocial variables that influence RTW				
Thornthwaite et al. 2017 [38]		AU		20		Non-specific work-related injury or illness; 60% male		Individual interviews (semi-structured)		Perceptions and experiences of injured workers interactions with insurers and employers				

Review 2: predictors of return-to-work (cohort and case control studies)														
Study	Country		Sample size		Population; type of injury/illness		Design		Injury onset to baseline			Follow-up period	Outcome	Predictors
Cohort studies														
de Vente et al. 2015 [40]	NL		71		Work-related stress complaints; 58% female; mean age in years (SD), 41.61 (9.48)		Prospective cohort		2 weeks to 6 months			13 months	RTW (full return at follow-up)	Co-worker and supervisor support
Jetha et al. 2017 [42]	AU		551		Work-related musculoskeletal pain/disorders; 48.8% male; 18–55+ years		Prospective cohort		> 10 days to 2 months since claim accepted			6 months	Sustained RTW (28 days or longer; baseline: 1–6 months post-injury; follow-up: 6 months after baseline)	Supervisor support; co-worker support
Kong et al. 2012 [43]	CN		335		Non-specific work-related injury or illness; 86% male; mean age in years (SD), 36.3 (9.7)		Retrospective cohort		Unknown			3–8 months	RTW (sustained for at least 3 continuous months during follow-up)	Family attitudes to RTW; personal feeling on social support for RTW
Li-Tsang et al. [45]	HK		75		Work-related repetitive strain injuries (age: 20–65 years)		Prospective cohort		< 3 years			–	RTW (employment status; 3.5 years post-injury)	Short Form 36 (SF-36) (social functioning)
Marois et al. 2009 [46]	CA		222		Work-related musculoskeletal pain/disorders; 59% male; mean age in years (SD), 39.1 (9.4)		Prospective cohort		Minimum 12 weeks			-	RTW (full-time or part-time, or those capable of RTW but unable due to obstacles unrelated to their injury or illness)	Loss of employment relationship; maintenance of contact between employee/employer; co-worker relationships; social isolation
Netterstrom et al. 2015 [47]	DK		223		Work-related common mental disorders; 19.7% male; mean age in years (SD), 44.2 (8.8)		Prospective cohort		Minimum 2 months			1 and 3 years	RTW (full time at baseline or follow-up)	Social support from colleagues and leader; low degree of justice; bullying
Reme et al. 2012 [48]	US		496		Work-related back pain; 58.1% male; mean age in years (SD), 37 (11.3)		Prospective cohort		< 14 days			3 months	RTW (work status at follow-up)	Workplace friendship; organisational support
Case–control studies														
Boot et al. 2014 [39]	CA		1561		Work-related back pain; 50.7% male; mean age in years (SD), 38.9 (11.1)		Case-control		–		12 months		RTW (any, including same/different employer, or modified work at follow-up)	Supervisor support
Holtedahl et al. 2007 [41]	NO		174		Non-specific work-related injury or illness; 56.8% male; mean age in years (SD), 43 (11)		Case-control		–		–		RTW (1–10 years post-injury; working full time, not working)	Social functioning (SF-36)
Lee et al. 2015 [44]	KR		2000		Non-specific work-related injury or illness; 84.3% male		Case-control		–		–		RTW (job retention, reemployment, unpaid family worker, self-employment; 24 months after terminating medical care)	Maintenance of relationship with employer
St-Arnaud et al. 2007 [49]	CA		1850		Work-related common mental disorders; 26% male; mean age in years (SD), 45 (8.3)		Case-control		–		–		RTW (not further specified; within 12 months)	Conflict with supervisors and/or co-workers; recognition of efforts
Watt et al. 2015 [50]	AU		110		Non-specific work-related injury or illness; 39% male; mean age in years (SD), 46.40 (11.06)		Case-control		–		–		RTW (durable: currently employed or previous employed > 12 months; non-durable: < 12 months)	Relationships with superior and colleagues; social support outside of the workplace

*NL* Netherlands, *AU* Australia, *CN* Canada, *HK* Hong Kong, *DK* Denmark, *US* United States of America, *NO* Norway, *KR* South Korea

#### Study Type

For Review Question 1, studies with a primary focus on qualitative methods, such as grounded theory, ethnography, and narrative analysis, were eligible for inclusion. For both Review Questions 2 and 3, studies with cohort, cross-sectional and case-control studies were eligible for inclusion, with Review Question 3 also including randomised controlled trials (RCT) and cluster-RCT designs as eligible. The inclusion of non-randomised designs in Review Question 3 was deemed necessary due to the difficulty in employing RCT designs within a worker’s compensation context. For all three review questions, case studies, review papers, and non-empirical papers (e.g. discussion, theoretical, opinion) were excluded.

### Information Sources and Search Strategy

Six electronic bibliographic databases: Medline (Ovid), PsycInfo (Ovid), EMBASE, the Cochrane Library, Scopus, and Family Health Database (ProQuest) were searched. The search was conducted in two broad categories: (1) Social/Community terms; and (2) Work/RTW terms. Terms within each category were combined with the ‘OR’ Boolean operator, with terms from the two categories combined with an ‘AND’ operator. The search terms were customised for each database (see “Appendix” for terms used in the Medline database). This strategy captured references that contained at least one term in each category. The search terms and strategy were checked with a Health Librarian. Searches were restricted to articles published in English from January 1, 2007 to February 15, 2018. The reference lists of relevant articles and recent systematic and narrative reviews were screened for additional publications.

### Data Management and Selection Process

Results from each database were combined into an EndNote library and duplicates removed prior to being imported into Rayyan QCRI [51] for screening. Two blinded reviewers (CW and SF) conducted the initial and full-text screens independently to determine whether articles met the eligibility criteria and how the article should be categorised for review. Based on the eligibility criteria, studies were assigned to one or more of the review questions, which focused on:

i. social barriers and facilitators for RTW identified by injured workers
ii. social predictors of RTW outcomes in injured workers, and
iii. interventions with social elements that targeted injured worker RTW outcomes.

**Fig. 1:**
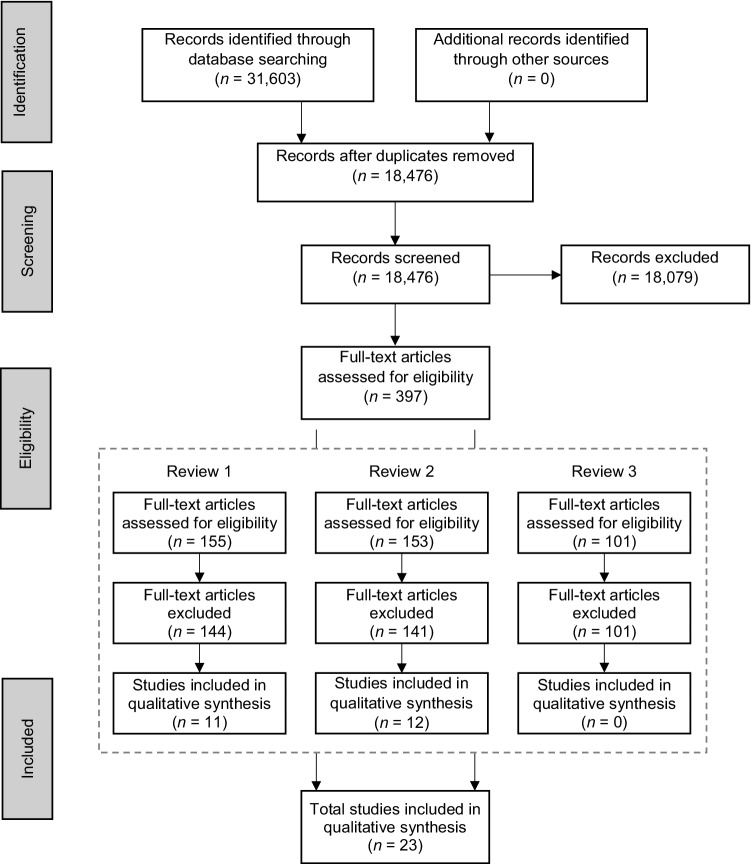
PRISMA flowchart for screening and selection of included studies

Initial screening was conducted on titles and abstracts, with articles of uncertain eligibility proceeding to the full-text screening. After initial screening, full-text screening commenced with the reviewers independently assessing each study against the inclusion and exclusion criteria. Disagreements between reviewers were resolved through discussion. The PRISMA flow diagram (see Fig. 1) provides further information.

### Data Collection

Data extraction was conducted using the full-text of each selected article. For all three review questions, data extraction included the following information (where available): source (author, journal, publication year), sample (age, sex, country); injury/illness (type, time from injury onset at baseline, time from injury onset at follow-up) employment (occupation type, work status); compensation type; and study design. In addition, some review-specific extractions items were included. For Review Question 1, data extraction included: social barriers to RTW (type, context), social facilitators of RTW (type, context) and RTW outcomes (type). For Review Question 2, data extraction included: social support/social integration (measure name, type, context), and RTW outcomes (measures, results). For Review Question 3, data extraction included: intervention details (name, type, duration, time after injury/illness, social element, other treatment elements, other participants), comparator, and RTW outcomes (measures, results).

A data extraction sheet developed by the authors was used to identify this information for each article. The two reviewers piloted the data extraction sheet with five randomly selected studies and the extraction sheet was refined as necessary. The extracted data was cross-checked by the reviewers and disputes were resolved were resolved through discussion.

### Risk of Bias

The included studies were evaluated for risk of bias and methodological quality using the Critical Appraisal Skills Programme (CASP) tools (https://casp-uk.net/casp-tools-checklists/). Two independent blinded reviewers assessed risk of bias, with disagreements resolved through discussion. As PRISMA guidelines note that reducing the risk of bias to a single numerical indicator can omit valuable information [52, 53], the full methodological components assessed for risk of bias are provided in Table 2. Based on these components, studies were ranked in quality based on the number of areas of potential risk. Studies where no risk of bias was detected were considered *high quality*, those with 1–2 areas of risk considered *medium quality*, and those with 3 or more areas of bias deemed *low quality*. Due to the small number of studies identified for inclusion in each review, low quality studies were not excluded, but were instead interpreted with caution.

**Table 2:** Quality appraisal information using CASP tools for all included studies

Review 1: facilitators of and barriers to return-to-work (qualitative studies)									
	Aims clearly stated	Appropriate method	Appropriate design to address research aims	Appropriate recruitment strategy	Data collection addressed research issue	Relationship between researcher/participants adequately considered	Ethical issues taken into consideration	Sufficient data analysis rigor	Clear statement of findings
Bunzli et al. 2017 [28]	✓	✓	✓	✗	✓	✗	✓	✓	✓
Buys et al. 2017 [29]	✓	✓	✓	✓	✓	✗	✓	✓	✓
Cheng et al. 2011 [30]	✓	✓	✓	✓	✓	✗	✓	✓	✓
Kosny et al. 2012 [31]	✓	✓	✓	✓	✓	✓	✓	✓	✓
Lysaght et al. 2008 [32]	✓	✓	✓	✓	✓	✗	✓	✓	✓
MacEachen et al. 2007 [33]	✓	✓	✓	✓	✓	✗	✓	✓	✓
Mansfield et al. 2014 [34]	✓	✓	✓	✓	✓	✗	✓	✓	✓
Mullen et al. 2015 [35]	✓	✓	✓	✓	✓	✓	✓	✓	✓
Norland et al. 2013 [36]	✓	✓	✓	✓	✓	✓	✗	✓	✓
Soklaridis et al. 2010 [37]	✓	✓	✓	✓	✓	✗	✓	✓	✓
Thornthwaite et al. 2017 [38]	✓	✓	✓	✗	✓	✓	✓	–	✓

Review 2: predictors of return-to-work (cohort and case control studies)									
Cohort	Clear focused issued	Appropriate cohort recruitment	Exposure accurately measured	Outcome accurately measured	Important confounding factors identified	Important confounding factors accounted for	Follow-up complete enough	Follow-up long enough	**–**
de Vente et al. 2015 [40]	✓	✓	✓	✗	✓	✓	✓	✓	
Jetha et al. 2017 [42]	✓	✓	✗	✗	✓	✓	✗	✗	
Kong et al. 2012 [43]	✓	✓	✗	✓	✓	✓	✓	✓	
Li-Tsang et al. [45]	✓	✗	✓	-	✓	✗	✓	✓	
Marois et al. 2009 [44]	✓	✓	✓	✗	✓	✓	–	–	
Netterstrom et al. 2015 [47]	✓	✓	✓	✓	✓	✓	✓	✓	
Reme et al. 2012 [48]	✓	✓	✓	✓	✓	✗	✓	✓	
Watt et al. 2015 [50]	✓	✓	✗	✗	✗	✗	✗	–	

Case control	Clearly focused issued	Appropriate method	Appropriate cases recruitment	Appropriate control selection	Exposure accurately measured	Important confounding factors identified	Important confounding factors accounted for	**–**	**–**
Boot et al. 2014 [39]	✓	✓	✓	✓	✓	✓	✗		
Holtedahl et al. 2007 [41]	✓	✓	✓	✓	✗	✗	✓		
Lee et al. 2015 [46]	✓	✓	✓	✓	✓	✗	✗		
St-Arnaud et al. 2007 [49]	✓	✓	✓	✓	–	✓	✗		

✓ = Criteria was met. ✗ = Criteria was not met. – = not eligible

## Results

Due to the heterogeneity across studies, a narrative synthesis approach was used to collate the findings in text, with tabular presentation of data, where appropriate. For all three review questions, findings are reported for all eligible studies, irrespective of bias.

### Study Selection

The search of databases provided a total of 31,603 articles. After duplicate removals 18,476 articles remained. Of these, 18,079 were removed after title and abstract screening, as they did not meet the eligibility criteria (e.g., different populations, outcomes). During this screening, articles were assigned to their relevant review question. For Review Question 1, there were 11 studies identified for inclusion. In Review Question 2, 12 studies were identified for inclusion. However, for Review Question 3, no studies were identified as eligible, therefore, results are not presented for this review question. For further details on the screening process see Fig. 1. No additional studies were identified by checking references of included articles.

### Characteristics of Included Studies

Included studies used a range of designs. To address Review Question 1, thematic analysis was used in four studies, grounded theory was used in three studies, two studies used focus groups, and one study each used naturalistic and constant comparative approaches. Five studies were conducted in Canada, three in Australia, and one each in Sweden, China, and the USA. Overall, risk of bias was low for studies addressing this review question. The area with greatest risk of bias was lack of consideration/reporting regarding relationship between researcher and participants, with seven of eleven studies at risk of bias. Two studies were also at risk of bias regarding appropriate recruitment strategies and one study did not report adequate considerations for ethical concerns. For Review Question 2, seven studies used prospective cohort designs, four used case–control designs, and one had a retrospective cohort design. Three studies were conducted in Canada, two in Australia, one each in China, Denmark, Hong Kong, South Korea, the Netherlands, Norway, and the USA. Overall, all eligible studies for this review question, except for one, were at risk of bias on at least one criterion. The areas displaying the greatest risk of bias (refer to Table 2) across studies included accurate measurement of exposure and outcome, with four out of twelve studies at risk of bias, and accounting for important confounds, with six studies at risk. Further details regarding study characteristics are available in Table 1.

## Review 1

Eleven studies [26–36] were identified that explored injured worker perspectives on key social factors that influenced their RTW process. Eight of these studies [29, 30, 32–37] reported the presence of social facilitators, while nine reported social barriers.

### Social Facilitators

Overall, seven social facilitators in the RTW process were identified. Five facilitators were primarily workplace-based. These included contact and good communication, genuine care and concern, organisational trust, validation and belief in injury legitimacy, and relationships in the workplace. A further two facilitators involved factors identified by injured workers from non-work contexts; these were advocacy support and family support. Details of each facilitator are given below.

#### Contact and Good Communication

Regular contact and good communication with the employer was identified as a facilitator of RTW in two medium quality studies [29, 34]. In four medium quality studies, supervisor communication was found to be most important for injured workers’ expectations of the RTW process, and in assisting with the worker's informational and practical RTW needs [29, 32, 36, 37]. Being able to discuss the injury or illness was also found to be important when communicating with co-workers in one medium quality study [30].

#### Genuine Care and Concern

RTW was shown to be supported, in one high quality and two medium quality studies, when injured workers received genuine concern from their co-workers [32, 35, 36], and from supervisors in two medium quality studies [32, 37]. RTW was also benefited when workers felt they were ‘more than a number’ [33], or worked within an organisational culture of genuine care [29]; in one medium quality study each.

#### Organisational Trust

A culture of mutual trust in the work community was identified as a facilitator of RTW [33], including the sense that the injured worker was trusted [32] and able to trust their supervisors [36]. These findings were from studies of medium quality.

#### Validation and Injury Legitimacy

Injured workers reported that it was important that they did not feel judged for resuming lighter duties and that others’ expectations of them post-injury were modified appropriately [32, 34, 36, 37]. It was also critical that supervisors believed in the legitimacy of injured workers’ claims [32]. These findings were from studies of medium quality.

#### Advocacy Support

Injured workers who experienced mistreatment or obstacles in navigating the workers’ compensation or internal RTW systems benefitted from support from peers who were knowledgeable and experienced in the RTW process and were on their side [29, 33]. Family members also provided advocacy support when questions of injury or symptom legitimacy were raised [34]. These findings were from studies of medium quality.

#### Family Support

Support from families was identified as a facilitator of RTW in two medium quality studies [32, 34], with workers often mentioning the importance of practical assistance (e.g., driving the injured worker to work or appointments) as important rather than purely emotional support.

#### Relationships with Work Community

While few studies directly implicated a sense of belonging as a facilitator of RTW, the existence of socially supportive relationships between the injured worker, their co-workers and employers was considered a facilitator of RTW in one medium quality study [34].

### Social Barriers

Overall, five social barriers to the RTW process were identified. Four RTW barriers stemmed from the workplace, including poor communication and support, impersonal processes, a lack of organisational trust, and hostile reactions to the injury or being judged by others. Feelings of isolation or being excluded from work and non-work (i.e., family) contexts were also identified as a barrier to RTW.

#### Poor Communication and Support

Contact from the workplace (e.g., employer, supervisors) that was delayed, infrequent, or did not provide meaningful information on the claims or RTW process was perceived to be a barrier to RTW in one high quality and two medium quality studies [29, 31, 38]. A lack of ongoing support from supervisors or employers and negative interactions were also barriers in the RTW process in one high quality and five medium quality studies [29, 31, 32, 34, 36, 38].

#### Impersonal Process

While most injured workers felt supported by their supervisors, a perceived lack of emotional support made it difficult for injured workers to RTW [34]. Process-centred systems that de-personalised the injured worker were also perceived to be barriers [33, 34], due to a lack of individual focus and emotional support. These findings were from studies of medium quality.

#### Lack of Organisational Trust

A lack of trust in the employer or supervisor was a barrier to RTW process, especially in terms of workers experiencing doubts that their employer would act in their best interests [28, 29, 32]. These findings were from studies of medium quality.

#### Hostile Reactions and Judgement

Negative reactions to an injury were identified as a major barrier of RTW. Within the work community, these experiences included a lack of validation and recognition of the workers’ concern in two medium quality studies [32, 33], feeling devalued by co-workers and employers in one medium quality study [37], and the experience of judgement, questions of injury legitimacy, or suggestions of malingering as reported in one high quality and three medium quality studies [31, 34, 36, 38]. Co-workers who reacted with indifference or hostility towards the injured worker functioned as barriers to RTW in one high quality and three medium quality studies [29, 31, 32, 37]. Further, in one high quality and three medium quality studies, more severe reactions (victim blaming, harassment, and stigma) were reported, all of which made it difficult for the injured worker to RTW [31, 34, 37, 38].

#### Exclusion and Isolation

Social exclusion and isolation were identified as barriers in the RTW process. Injured workers reported in two high quality studies that a culture of isolation and exclusion of injured workers was sometimes encouraged by supervisors [31] and this led to feeling isolated or excluded by co-workers [31, 35]. Injured workers also reported feeling alienated from former sources of support, stating in one medium quality study that their families did not understand the difficulties associated with managing their work-related injury or illness [33].

## Review 2

Twelve studies were identified that examined social support or social integration as predictors of RTW outcomes (see Table 3). Of these, the majority focused on work-related social sources: one study examined organisational factors, seven examined co-worker factors, and seven examined supervisor factors. Three main predictors were distinguished across co-worker and supervisor: social support, response to injury, and relationships. Social factors outside of work settings were also considered, with two studies examining family factors, three examining social functioning, and one examining overall social support.

**Table 3:** Univariate and multivariate findings for Review Question 2

Construct	Studies measuring construct	How construct was measured	Univariate		Multivariate			Outcome	Controlled for
			Baseline	Follow up	Baseline		Follow up		
Organisational social factors									
Organisational support	Reme et al. 2012 [48]	Eight-item shortened version of the Perceived Organization Support Scale (7-point scale)	✓ (OR 0.88)	–		–	–	RTW (work status at 3 month follow-up)	–
Supervisor social factors									
Supervisor support	deVente et al. 2015 [40]	Subscale of Job Content Questionnaire (4-point scale)	✕	–		–	–	RTW (full return for at least 1 month, within 13 months post-injury)	–
	Jetha et al. 2017 [42]	Three questions were posed on supervisor support (five point scale)	✓ NR	✕		✕	✕	Sustained RTW (28 days or longer; baseline: 1 to 6 months post-injury; follow-up: 6 months after baseline)	Age, gender, injury type, time since injury, work-context factors + *Reaction factors* Follow up: +RTW at baseline, time off
	Netterstrom et al. 2015 [47]	Subscale of Copenhagen Psychosocial Questionnaire (COPSOQ) (4-point scale)	✓ (r = − .147)	–		✓ NR ✕	–	RTW (full time; baseline: 1 year; follow-up: 3 years)	Age, gender, marital status, and occupational position Severity of illness
	Watt et al. 2015 [50]	Questionnaire: Experience and Evaluation of Work (QEEW)—relationships with superiors subscale (4-point converted to 0–100)	✓ (η^2^ = 0.14)	–		–	–	RTW (durable: currently employed or previous employed > 12 months; non-durable: <12 months)	–
Supervisor injury response	Boot et al. 2014 [39]	Interview question based on literature (multi-choice) ‘Please tell me whether any of the following list of reactions that your supervisor may have had to your accident/injury apply to your case’ (eight items yes–no)	✓ (OR: 1.7)	–		–	–	RTW (any, including same/different employer, or modified work at follow up; follow-up: 12 months)	–
	Jetha et al. 2017 [42]	Given eight reaction types and asked if applicable to experience (yes–no)	✓ (*w* = 0.17)	✓ (*w* = 0.18)		✓ (OR 1.6) *✓* (OR 2.3)	✓ (OR 1.6) *✕*	Sustained RTW (28 days or longer; baseline: 1 to 6 months post-injury; follow-up: 6 months after baseline)	Age, gender, injury type, time since injury, work-context factors + *Support factors* Follow up: +RTW at baseline, time off
Relationship w/employer	St Arnaud et al. 2007 [49]	Developed questionnaire that asked related questions	✓ (PR 1.04)	–		✓ (PR 1.00)	–	RTW (not further specified; within 12 months)	Age, gender, job type, working conditions, work-related factors
Maintenance of relationship w/employer during time absent	Lee et al. 2015 [44]	Data from the first PSWCI, published in June 2014	✓ (*w* = 0.21)	–		✓ (OR 1.79)	–	RTW (job retention, reemployment, unpaid family worker, self-employment; 24 months after terminating medical care)	Age, gender, education, smoking, alcohol, income, registered as disabled, occupational characteristics, physician-related factors, employer-related factors
Co-worker social factors									
Co-worker social support	deVente et al. 2015 [40]	Subscale of Job Content Questionnaire (4-point scale)	✕	–		–	–	RTW (full return for at least 1 month, within 13 months post-injury)	–
	Jetha et al. 2017 [42]	Five questions were posed on co-worker support (5 point scale)	✓ NR	✓ NR		✕	✕	Sustained RTW (28 days or longer; baseline: 1 to 6 months post-injury; follow-up: 6 months after baseline)	Age, gender, injury type, time since injury, work-context factors + *Reaction factors* Follow up: +RTW at baseline, time off
	Netterstrom et al. 2015 [47]	Subscale of Copenhagen Psychosocial Questionnaire (COPSOQ) (4-point scale)	✓ (r = − .141)	–		✓ NR X	–	RTW (full time; baseline: 1 year; follow-up: 3 years)	Age, gender, marital status, and occupational position Not specified
	Watt et al. 2015 [50]	Questionnaire on the Experience and Evaluation of Work (QEEW): relationships with colleagues subscale (4-point converted to 0-100)	✓ (η^2^ = 0.07)	–		–	–	RTW (durable: currently employed or previous employed > 12 months; non-durable: <12 months)	–
Co-worker injury response	Jetha et al. 2017 [42]	Given five reaction types and asked if they applied to their experience (yes–no)	✕	✓ (*w* = 0.10)		✕	✕	Sustained RTW (28 days or longer; baseline: 1 to 6 months post-injury; follow-up: 6 months after baseline)	Age, gender, injury type, time since injury, work-context factors + *Support factors* Follow up: +RTW at baseline, time off
Relationship w/colleagues	Marois et al. 2009 [46]	Existing database. Used semi-structured interview and self-administered questionnaires	✕	–		–	–	RTW (full-time, part-time, or capable of RTW but unable due to obstacles unrelated to work injury or illness)	–
	Reme et al. 2012 [48]	Workplace Friendship Scale; Six items (7-point scale)	✕	–		–	–	RTW (work status at 3 month follow-up)	–
	St Arnaud et al. 2007 [49]	Developed questionnaire that asked related questions	✕	–		✕	–	RTW (not further specified; within 12 months)	Age, gender, job type, working conditions, work-related factors
Non-work social factors									
Family/friends	Kong et al. 2012 [43]	Self-reported family’s attitude on RTW (4—response multi-choice; less than positive, no comment, positive, unknown)	✓ NR ✓	–		✓ (HR: 4.0)	–	RTW (sustained for at least 3 continuous months during follow-up: 3 to 8 months) Absence Duration	Gender, marital status, residential status, enterprise ownership when injury occurred, job position, working years pre-injury, monthly salary pre-injury, injury body part, injury nature, communication with employers, and occupational rehabilitation exercises
	Watt et al. 2015 [50]	Multidimensional Scale of Perceived Social Support (MSPSS) (12-items; 7-point scale; overall score)	✕	–		–	–	RTW (durable: currently employed or previous employed > 12 months; non-durable: <12 months)	–
Social functioning	Boot et al. 2014 [39]	Short Form Health Survey (SF-36)—social functioning subscale, range (0–100)	✕	–		–	–	RTW (any, including same/different employer, or modified work at follow up; follow-up: 12 months)	–
	Holtedahl et al. 2007 [41]	Short Form Health Survey (SF-36): Social functioning subscale (0-100)	✓ (*d* = 0.93)	–		–	–	RTW (post-injury: working full time, not working)	–
	Li Tsang et al. 2007 [45]	Short Form Health Survey (SF-36): social functioning subscale (0-100)	–	–		✓ NR	–	RTW (employment status; 3.5 years post-injury)	Sex, age, educational level, type of injury
Overall social factors									
Overall social support	Kong et al. 2012 [43]	Self-reported feelings on Social support 4-response multi-choice (very satisfied, satisfied, dissatisfied, no comment)	✓ (*w* = *0.24)* ✕	-		✓ (HR: 2.1)	–	Sustained RTW (3 continuous months during follow-up) Absence duration	Gender, marital status, residential status, enterprise ownership when injury occurred, job position, working years pre-injury, monthly salary pre-injury, injury body part, injury nature, communication with employers, and occupational rehabilitation exercises

Univariate analyses only included the social predictor and RTW variable. Multivariate analyses controlled for other variables (social and non-social)

OR = Odds Ratio; NR = Effect size not reported; r = Correlation; η^2^ = Partial eta squared; *w* = *Phi, PR* = Prevalence Ratio; HR = Hazard Ratio; d = Cohen’s d

✓ = significant positive relationships were found, ✕ = relationships were non-significant, – = relationship was not examined

### Organisational Social Factors

The role of organisational support for RTW was underrepresented in the literature. Only one medium quality study [48] investigated organisational support, which was found to predict RTW.

### Supervisor Social Factors

Supervisor social factors included supervisor social support, supervisor reaction to injury and employer–worker relationship. Moderate support was found for the role of the employer–worker relationship through two medium quality studies [44, 49]. In these studies, injured workers’ relationship with their employer and maintenance of this relationship after injury predicted RTW status [44, 49]. This effect remained significant when explored through multivariate analyses.

Supervisor social support was also examined in four studies [40, 42, 47, 50]. Two studies provided mixed support for RTW status; a high quality study [47] indicated supervisor support was a significant univariate predictor of RTW status, although mixed findings emerged in multivariate analyses, while a medium quality study [40] reported no significant finding of supervisor support. Two low quality studies provided similarly mixed support for SRTW; in one study [50], supervisor support predicted 12-month SRTW, while another [42] produced mixed results.

Supervisors’ response to injury significantly predicted RTW status in two studies [39, 42]. One medium quality study [39] found supervisors’ response to injury predicted RTW status, while one low quality study [42] found this predicted 1-month SRTW in a univariate analysis (with multivariate analyses producing mixed findings). In both supervisor social support and response to injury, the mixed findings and risk of bias makes interpreting the relationship between supervisor support and RTW difficult.

### Co-worker Social Factors

Co-worker social factors included co-worker social support, co-worker reaction to injury and relationship with co-workers. Four studies examined the influence of co-worker social support on RTW outcomes [40, 42, 47, 50]. Co-worker social support was predictive of RTW status in one high quality study [47], although findings were mixed when examined through multivariate analyses. The SRTW outcome yielded similarly mixed findings, with two low quality studies [42, 50] offering some support for co-worker social support, while a medium quality study found no significant relationship with 4-week SRTW [38]. The low quality studies found co-worker support predicted 1-month SRTW at baseline and 6-month follow-up (although this was non-significant in multivariate analyses) [42], and at 12-month SRTW in the second study [50].

Only one low quality study examined co-worker’s response to injury [42]. Findings from this study were mixed: 1-month SRTW was not predicted by co-workers’ response to injury at the baseline assessment, but this was significantly predicted at a 6-month follow-up in both univariate and multivariate analyses [42].

Three medium quality studies examined the influence of relationships with co-workers [46, 48, 49]. In all three studies, the quality of co-worker relationships was not a significant predictor of RTW outcomes.

### Non-work Social Factors

There were two studies that examined the influence of social support from family and/or friends on SRTW [43, 50], while another three studies [39, 41, 45] examined social functioning on RTW status. For social support, one medium quality study [43] found family attitudes toward RTW were significant predictors of 3-month SRTW in both univariate and multivariate analyses, while a low quality study [50] did not find social support from family, friends, and significant others to be predictive of 12-month SRTW. RTW status was not examined as an outcome in any studies examining social support from family/friends.

Mixed support emerged for social functioning of injured workers as a predictor of RTW status from three medium quality studies [39, 41, 45]. Social functioning was defined in all three studies as the level of interference the injured worker experienced with regular social activities, such as visiting with friends or relatives. Higher social functioning was found among individuals who had experienced RTW compared to those who had not [41], while social functioning was also found to be a significant predictor of RTW in multivariate analyses [45]. However, a third study found that social functioning did not predict RTW status 12 months following injury [39].

### Overall Social Factors

The single medium quality study that examined overall social support as a predictor of SRTW found that satisfaction with received social support significantly predicted 3-month SRTW in both univariate and multivariate analyses [43]. No included studies examined overall social support on RTW status.

## Discussion

The current review was the first to systematically examine the impact of social factors on RTW in individuals with work-related injuries. The strongest evidence emerged from qualitative studies, which reported on a variety of social facilitators and barriers. However, evidence was also found to suggest that some social factors could significantly predict RTW outcomes in quantitative studies. Despite this emerging evidence for the facilitative role that social support and integration can provide in the RTW process, no social interventions were identified as eligible for inclusion and as such, it was not possible to address Review Question 3.

### Review Question 1: Social Factors as Facilitators and Barriers to RTW

In total, seven facilitators and five barriers were identified as important in the RTW process by injured workers. The majority of these social factors were both facilitators (when present or positive) and barriers (when absent or a negative experience). As such, there were five overarching themes that encompassed the identified social factors: (1) contact and communication, (2) person-centred approach, (3) mutual trust, (4) reaction to injury, and (5) social relationships and integration.

The themes most commonly reported were *contact and communication* and *reaction to injury*. To support the RTW process, moderate evidence across medium to high quality studies suggested that communication needed to be timely, ongoing, and informative. Moderate evidence across medium to high quality studies also suggested that reactions to the injury needed to be positive to ensure that the worker felt validated, rather than judged, and demonstrate that colleagues believed in the legitimacy of their injury. In one study, negative reactions to injury were reinforced by supervisor actions, which communicated to co-workers that it was acceptable to treat injured workers poorly [31]. This created barriers in the RTW process, demonstrating the importance of supervisors and the wider organisational culture for the level of social support provided by the work community. The second largest theme was the *person-centred approach*, where moderate support was found that authentic and genuine care and a human element to the workers’ compensation and RTW system were important factors for RTW across medium to high quality studies. Additional evidence for this was provided within the theme of *trust*, where there was moderate indication across medium quality studies that the worker needed to feel trusted and feel that their supervisor could be trusted to act in their best interest. In addition to these forms of social support, injured workers identified *social relationships and integration* as beneficial for achieving a smooth RTW process, with moderate support across medium to high quality studies. Injured workers with supportive relationships in their work and home communities found this facilitated RTW, while isolated or excluded workers found the RTW process more challenging.

The social facilitators and barriers to RTW also appeared consistent with the five forms of social support proposed by Brissette et al. [14]. Early research by House [54] and Barrera [55] posited four forms of social support, which included emotional (genuine concern and trust), instrumental (work adjustments or practical assistance), appraisal (feedback and validation), and informational (help navigating the system or advocacy) support. However, Cohen and colleagues’ addition of companionship support also appears relevant to RTW, as social relationships had a facilitative effect and exclusion and isolation were barriers to RTW. The findings for companionship support are consistent with previous reviews outside work rehabilitation contexts, which found that social contact, loneliness and social relationships have an important influence on health-related outcomes [5, 6]. It was also noteworthy that in addition to negative reactions, a lack of support or indifferent reactions were raised as barriers. This is consistent with findings from Yang and colleagues [56] suggesting a continuous dose–response effect for social factors (i.e., increasing social connection results in more positive health outcomes). This suggests that further emphasis should be placed on increasing positive reactions to injury rather than simply reducing negative reactions.

### Review Question 2: Social Factors as Predictors of RTW Outcomes

As with past reviews examining social predictors for RTW, few eligible studies were identified that examined social factors as predictors of RTW among injured workers [25]. Of those included, 10 of 12 studies found at least one social factor predicted RTW outcomes. However, the high degree of inter-study heterogeneity and lack of consensus in the measurement of social factors [25, 57] limits the strength of conclusions that can be drawn on the impact of social factors overall.

While strong conclusions cannot be drawn for social factors overall, support was found for the importance of individual social facilitators. Consistent with Review 1, there was moderate evidence found for both reaction to injury and social integration as predictors of RTW. Specifically, positive supervisor responses to injury predicted better RTW outcomes, suggesting that supervisor attitudes to and interactions with employees may be a key target for intervention within the work community. Maintaining good social functioning was also supported as a predictor of RTW outcomes, indicating that engaging injured workers in social activities with family, friends or other community groups may promote better RTW outcomes. This is similar to the behavioural component of social integration. In both cases, two medium quality studies supported these predictors, while for social functioning, one medium quality study did not find this relationship. Further research is needed to better understand the relationship between social support and social integration and RTW outcomes.

In terms of sustained RTW, social factors were also found to be a potentially important factor, with weak evidence found for co-worker social support as a predictor of improved sustained RTW, with two of three studies finding a significant relationship. This suggests that the influence of colleagues may be most apparent upon work return, where negative attitudes or hostility towards the injured worker may become apparent. However, as both studies supporting the role of co-worker social support for SRTW were at heightened risk of bias, further research is needed into this area. Overall, the current review indicates that both work and non-work communities may play important roles in the RTW process. This is consistent with suggestions [57] that multiple contexts (e.g., supervisor reactions, co-worker support, and social functioning and relationships) may need to be targeted to produce effective interventions for RTW.

Some support was also found for the role of other social factors in the RTW process (e.g., organisational, supervisor, co-worker and family support). However, while no study found social support or social integration to be detrimental to RTW outcomes, further research into these domains is needed. In particular, research into sickness absence has identified a range of social factors as helpful in the RTW process. This includes specific aspects of social support [58, 59], as well as social integration factors such as feeling welcomed back and sense of community [58].

### Review Question 3: Interventions

Review 3 asked ‘Are interventions focused on social support and social integration in work and non-work-related contexts effective in increasing RTW outcomes for individuals with work-related injuries?’. While some interventions have been conducted with social aspects, such as communication or group-based activities (e.g., [60–63]), these interventions were not directed towards individuals who had received a work-related injury. Further research should be conducted examining the efficacy of such interventions for work-related or compensable injuries as these workers have been found to experience longer delays in work return than workers with non-compensable injuries [17]. The design of future interventions should take into account the evident importance of social factors and target these factors to enhance RTW outcomes. Based on the findings in Review 1, interventions can be tailored for work or non-work contexts. Interventions tailored for non-work contexts are expected to improve RTW in situations where there has been a breakdown of relationships in the workplace or when inadequate support is provided.

### Strengths and Limitations of the Review

The current review was conducted within PRISMA guidelines using an established measure of critical appraisal (CASP) in the evaluation of the included studies. At present, this review is the first to examine social support and integration on the RTW process. However, the current review suggests that social factors are often neglected in the investigation of injured workers’ recovery and rehabilitation. The tripartite approach to the review allowed the contributions of qualitative and quantitative studies to be examined in an emerging field of interest. This review provides a timely summary of the available evidence for improving injured workers’ RTW experience, which can be used as a starting point to drive research into a currently understudied area.

There were limitations in both the available literature and the current review. In particular, there is a paucity of research into the influence of social factors during the recovery of work-related injuries. This was most apparent for Review Question 3, where no eligible interventions were identified. In Review 2, there was also a relative lack of studies using prospective cohort designs, and no eligible studies using RCT designs, limiting the ability to infer causality from predictive social factors. Overall, the small number of eligible studies for review suggests that this field is either under researched or constrained by publication bias. This is discrepant with the strong emphasis on the biopsychosocial model found in rehabilitation literature [1, 42]. Future reviews could assess, and potentially overcome, publication bias through examination of the grey literature. In addition, due to the limited literature, all eligible studies were included, regardless of the risk of bias identified. The review was also limited by the heterogeneity of the examined literature, with studies using a variety of RTW outcomes and follow-up periods, as well as a wide range of study designs and omnibus analyses. These considerations may limit the strength of the conclusions drawn. The choice to summate evidence from individuals with a work-related injury or illness, without limiting the review to a specific population (e.g., lower back injury, common mental disorders), was made to obtain a global indication of the social factors that are broadly relevant to RTW. However, this may have obscured within-population differences in the importance of different types of social support for RTW.

### Conclusions and Recommendations

The current review provided the first systematic investigation into the role of social support and social integration in RTW outcomes for work-related injuries or illnesses. Evidence was found from both qualitative and quantitative studies for the beneficial effect of social support in the RTW context, although this support came from a relatively small and heterogeneous number of studies. Review 1 identified five themes surrounding the social factors that workers felt were important to their RTW: (1) contact and communication, (2) person-centred approach, (3) mutual trust, (4) reaction to injury, and (5) social relationships and integration. The presence of these social factors was reported to facilitate RTW, while their absence was a barrier to RTW. There was moderate support, obtained from Review 2, for reaction to injury, social integration and social functioning as predictors of RTW, and weak support for co-worker support in predicting SRTW. Support for other social elements was mixed and inconclusive. Review 3 could not be conducted, with no studies meeting the eligibility criteria for interventions that targeted social factors for improved RTW outcomes.

The authors provide two recommendations to enhance future understandings of the contribution of social support in an injured worker population. First, the current lack of consistency in the measurement and conceptualisation of social factors hinders the synthesis of research in this area. The studies used a range of measures of social factors, usually taken from larger, generalised multidimensional instruments of organisational factors. It is, therefore, recommended that a comprehensive, standardised tool be developed for the measurement of these social factors. The effective conceptualisation and measurement of social factors in the recovery and RTW process is expected to indeterminably advance social research and inform practice in the field of occupational rehabilitation. Second, future research into the recovery and RTW outcomes of injured workers should endeavour to include the influence of social factors, especially when designing or evaluating interventions. The views of other stakeholders such as employers, direct supervisors, and co-workers regarding the influence of social factors on injured workers could also be examined. This will allow better access to evidence-based strategies when workplace rehabilitation practitioners consider social factors in the recovery and RTW process and facilitate greater recognition of the “social” component within the biopsychosocial model.

## Appendix

### Ovid-Medline Search

((sociodemographi*:ab,tl or “socio-demographi*”:ab,tl or biopsychosocial:ab,tl or “bio-psycho-social”:ab,tl or “bio-psychosocial”:ab,tl or “biopsycho-social”:ab,tl or psychosocial:ab,tl or “psycho-social”:ab,tl or socia*:ab,tl or alienat*:ab,tl or isolat*:ab,tl or lonel*:ab,tl or belon*:ab,tl or communi*:ab,tl or suppor*:ab,tl or peer*:ab,tl or colleagu*:ab,tl or superviso*:ab,tl or coworke*:ab,tl or “co-worke*”:ab,tl or families:ab,tl or family:ab,tl or familial:ab,tl or frien*:ab,tl, or mento*:ab,tl or interpersonal:ab,tl or spous*:ab,tl or partne*:ab,tl) and (“return to work”:ab,tl or “return-to-work”:ab,tl or “back to work”:ab,tl or “back-to-work”:ab,tl or “worker compensation”:ab,tl or “workers compensation”:ab,tl or “worker’s compensation”:ab,tl or “injured worker”:ab,tl or “sick leave”:ab,tl or “disability leave” :ab,tl “injured employee*”:ab,tl or “work related injury”:ab,tl or “work-related injury”:ab,tl or “sickness leave” :ab,tl or “work return”:ab,tl or “disability management” :ab,tl or “work injur*”:ab,tl or “workplace injur*”:ab,tl or “occupational injur*”:ab,tl or “occupational diseas*”:ab,tl or “occupational illness” :ab,tl or “occupational acciden*” or “industrial injur*”:ab,tl)).
